# Sender–receiver systems and applying information theory for quantitative synthetic biology

**DOI:** 10.1016/j.copbio.2014.08.005

**Published:** 2015-02

**Authors:** Diego Barcena Menendez, Vivek Raj Senthivel, Mark Isalan

**Affiliations:** 1 EMBL/CRG Systems Biology Research Unit, Centre for Genomic Regulation (CRG), Dr. Aiguader 88, 08003 Barcelona, Spain; 2 Universitat Pompeu Fabra (UPF), Barcelona, Spain; 3 Department of Life Sciences, Imperial College London, London SW7 2AZ, UK

## Abstract

Sender–receiver (S–R) systems abound in biology, with communication systems sending information in various forms. Information theory provides a quantitative basis for analysing these processes and is being applied to study natural genetic, enzymatic and neural networks. Recent advances in synthetic biology are providing us with a wealth of artificial S–R systems, giving us quantitative control over networks with a finite number of well-characterised components. Combining the two approaches can help to predict how to maximise signalling robustness, and will allow us to make increasingly complex biological computers. Ultimately, pushing the boundaries of synthetic biology will require moving beyond engineering the flow of information and towards building more sophisticated circuits that interpret biological meaning.

## Highlights

• Synthetic biologists are building diverse sender–receiver (S–R) systems.
• Information theory allows a quantitative analysis of S–R function.
• S–Rs can employ direct contact or extracellular signal diffusion.
• S–R channels can even occur within single proteins.
• Applications include distributed, parallel multicellular computers.

## Graphical abstract

**Figure:**
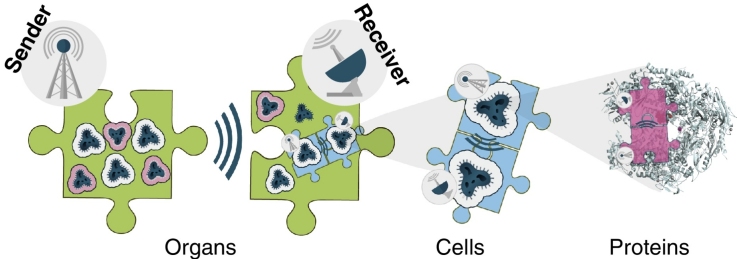

**Current Opinion in Biotechnology** 2015, **31**:101–107

This review comes from a themed issue on **Analytical biotechnology**

Edited by **Hadley D Sikes** and **Nicola Zamboni**

For a complete overview see the Issue and the Editorial

Available online 1st October 2014

**http://dx.doi.org/10.1016/j.copbio.2014.08.005**

0958-1669/© 2014 The Authors. Published by Elsevier Ltd. This is an open access article under the CC BY license (http://creativecommons.org/licenses/by/3.0/).

## Introduction

There is an intrinsic drive for biological entities to cooperate and coordinate responses to environmental queues. From DNA replication to bacterial quorum sensing, through to bird flock behaviours, and even in human economical structures, biological systems organise behaviours via communication. Signals by themselves do not usually contain any meaning, i.e. supplying useful patterns, materials or energy. Rather, meaning appears only when the agents involved in communication interpret the information. But how can we in the life sciences quantify this information?

The mathematical formulation of communication systems and information was laid down by Claude Shannon in a landmark 1948 paper [1]. Shannon showed that axiomatic rules describe and predict communication between a sender and a receiver, establishing limits in mutual information transfer imposed by the channel in which a message is transmitted. The beauty of Shannon's work is that it applies to any system that can be abstracted to a sender–receiver (S–R) topology.

S–R systems use the ‘bit’ as the unit of information, and this is the ratio of the probability of a state, given that a signal has been received, versus the probability of a state without a signal. In other words, the quantity of information in a signal can be measured by the shifts in state probabilities. However, some researchers argue that it is equally important to have a measure for the context or ‘meaning’ of a signal as well as the quantity [2].

**Figure 1:**
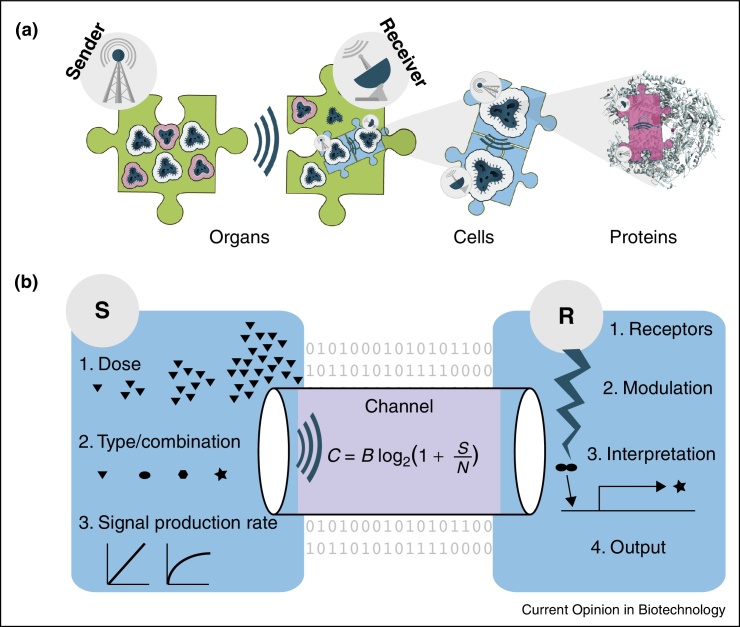
Sender–Receiver (S–R) systems occur at all levels of biology. **(a)** The schematic visualises these layers of communication as a multi-level jigsaw puzzle, working between factors such as proteins, cells and organs. In principle, Shannon's information theory can be used to quantify the information flow in all such S–R systems. The Proteins image, Alpha-Amanitin–RNA polymerase II complex, is licensed under Public domain via Wikimedia Commons. **(b)** Inputs from the sender (S) vary in terms of dose (e.g. chemical concentration), type of biomolecule (e.g. AHL, volatile Aldehyde, Dopamine, or even DNA fragments) and the rate of production. The channel is the medium of information transfer. The channel capacity *C* (*measured in bits per second*) is modulated by the equation shown; bandwidth *B* is the range of frequency allowed by the channel (the change in concentration of molecules; Hz) and *S* and *N* are signal and noise respectively. The receiver (R) mediates signal reception via cognate receivers like cell surface receptors. A modulation system like a cell signalling pathway links the signal to the interpreter (e.g. a responsive promoter for gene expression) resulting in extraction of the ‘meaning’ in the signal. The outputs, such as gene expression, are measured relative to space, time and input dose responses.

In this review, we will focus on studies relating to S-R systems with cells and biomolecules as the information processing agents. We will outline recent developments that allow biologists to quantify signalling, and how this is giving us a first glimpse into Shannon's predictions in biological systems (Figure 1).

First, we will look at S–R systems where the signal is transmitted through direct contact (intra- or inter-cellular). Next we will consider systems with signal transmission through external media, including diffusion processes, complex multicellular information processing and pattern formation. The most important advance is that new studies are using the tools of synthetic biology to build S–R systems from the bottom-up. While synthetic biologists aim to harness the power of biological systems, the insights we gain into cellular communication may allow us to move from the concept of information into engineerable definitions of ‘meaning’.

## Single proteins contain internal information channels

**Figure 2:**
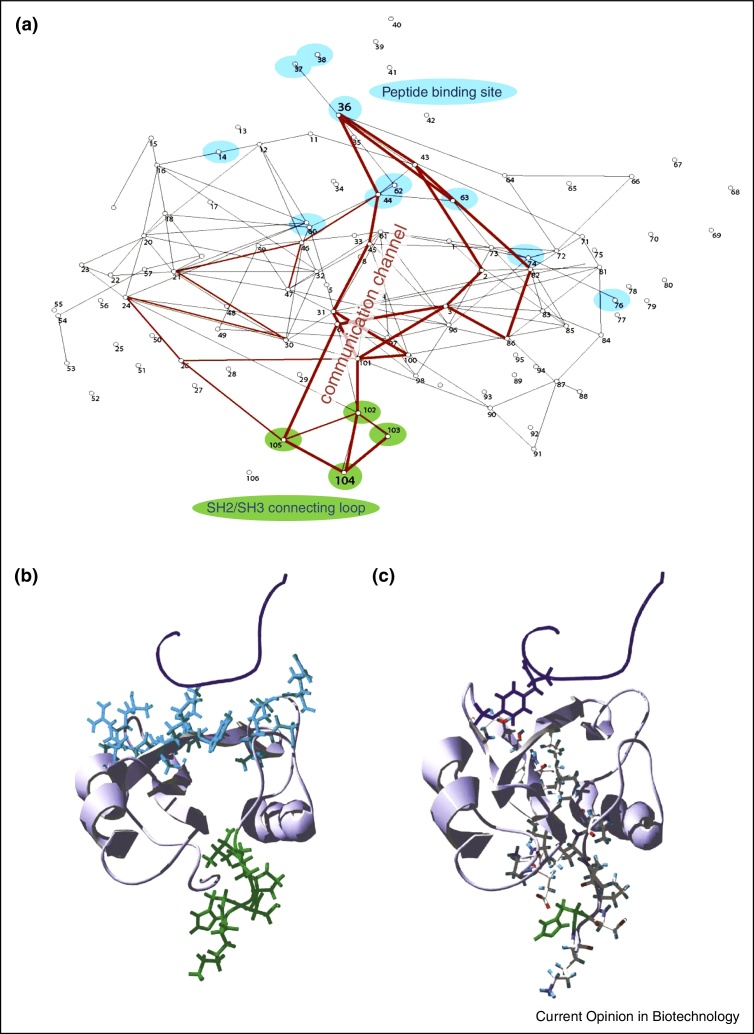
An information channel within a single protein. **(a)** A recent study [3^••^] visualised the communication channel within the Fyn SH2 domain, linking a peptide binding site (blue), and an SH2/SH3 connecting loop for Fyn kinase (green). The change in mutual information upon binding is measured between each pair of residues (white nodes). Adjacent residue pairs with significant changes in mutual information are represented as black or red lines. The largest changes, shown in red, are observed between residues forming a connected path from the peptide binding region to the connecting loop region. Thus, information theory reveals the major communication path. **(b)** and **(c)** are two structural views, highlighting the positions of the residues involved in the binding region and loop region (b) or the communication channel (c). The peptide (including the phosphorylated tyrosine) is in dark purple (top), the peptide binding site is in blue and the connecting loop is in green (bottom). Images kindly provided by Dr. Jesper Ferkinghoff-Borg and Dr. Joost Schymkowitz.

Perhaps the simplest biological S–R system involves the allosteric communication of domains within a single protein. In a remarkable study, researchers visualised the communication channel within the Fyn SH2 domain, showing a noisy protein conformation ‘wire’ linking the two sides of the protein [3^••^] (Figure 2). By combining structural modelling and information theory, they showed how this channel transferred SH2 binding information towards the SH3 and kinase domains. Going one layer of complexity further, they later explored Shannon's mutual information transfer in a protein signalling cascade: the p27 regulatory pathway [4]. By quantifying engineering properties, such as channel noise and channel capacity, they could identify protein concentrations for optimum switching and signalling. Applying information theory clearly has the potential to give us new quantitative insights in biology [5,6].

## Artificial stimulation of nervous systems to transmit information

Communication by direct contact occurs both within and between cells, and neurons were the first cells to be described as senders and receivers of information. Early experiments, such as stimulating and recording electrical signals through single neurons in the *Aplysia deplians* giant cell [7], eventually led to modern techniques in electrophysiology. Combined with recent genetic tools [8–10], and imaging techniques such as confocal fluorescence microscopy, fMRI BOLD (blood oxygenation level-dependent magnetic resonance imaging) and CLARITY [11], a full connectivity map of the brain is within our reach.

The development of optogenetics ([12], reviewed in [13]) allows stimulating a single neuron with light in one region of the brain. By stimulating the cortex, and measuring a distal receiver response in the thalamus, particular network behaviours have been observed, such as signalling delays [14]. It is fascinating to imagine how the application of quantitative information theory approaches to these S–R systems will reveal new insights into the transmission of thought.

Optogenetic techniques are also being used to map the neuronal networks responsible for locomotion, by targeting glutamatergic neurons [15,16]. It is possible, in principle, to stimulate spinal chord neurons (senders) to elicit a response in motor neurons (receivers). Thus, the tantalising prospect of being able to programme movement genetically emerges [17].

Understanding neural S–R systems, and their reciprocal signalling with the body, is already opening new fields in medicine. Murakami and colleagues [18] demonstrated that inducing electrical signals in mouse soleus muscles can open the brain–blood barrier to immune system T cells. Furthermore, Torres-Rosas *et al.* activated the sciatic nerve and dramatically reduced the levels of autoinflamatory cytokines in a sepsis model mouse [19^•^]. Engineering electrochemically-coupled S–R systems is only just beginning and has great potential for both biomimetics and synthetic neural networks.

## Developmental signalling can occur with direct cell-cell contacts

Developmental patterning provides us with a huge range of S–R systems to explore, and direct cell-cell communication is exemplified by the Notch–Delta system found in most multicellular organisms (reviewed in [20]). By acting in both cis and trans, these cell membrane receptors directionally shape pattern formation [21]. The receptors are providing new tools for synthetic biology, such as engineering trigger waves for intercellular information propagation, by transplanting Notch–Delta systems into naive cells [22].

The gap between nearby intercellular and distal multicellular communication is filled by organisms such as the fungus *Physarum Polycephalum*, which communicates with long protoplastic tubes to send signals between cells [23]. Strikingly, the organisation of tubes optimises resource distribution [24,25], and the electric potential recorded between joined cells resembles brain waves [26]. Information transfer in *Physarum* involves multiple mechanisms: feeding protoplastic arms with fluorescent beads has revealed a peristaltic mechanism for signal transport [27]. This capability has been translated into computer algorithms to model dynamical transport networks [28,29]. Furthermore, *Physarum* is a robust organism which can grow on many different substrates, making it a good candidate for development of synthetic biosensors [30]. Overall, such systems may provide an intriguing scaffold for engineering contact-based S–R systems and studying them on a quantitative basis.

## Synthetic bacterial S–R systems employ signal diffusion for patterning

Contactless S–R systems, with diffusing biochemical signals, have been a major focus of research in synthetic biology and have been reviewed extensively elsewhere [31,32]. The first example of a synthetic S–R system involved a pulse generating response in *E. coli* [33^•^]. Sender cells secreted the quorum-sensing signalling molecule acyl-homoserine lactone (AHL) while receiver cells activated a feed-forward transcription factor network to create a transient pulse of GFP expression. Thus, the simple diffusing signal created dynamic spatiotemporal patterns of gene expression. Later studies demonstrated elegant stripe or band-patterning systems, also using quorum-sensing signalling components [34^••^]. Quorum sensing S-R systems have even been coupled to cell motility [35^•^], thus achieving self-organisation of highly regular stripe patterns. Self-organising systems do not always need spatial S–R signalling, and a recent band-forming system relied entirely on a temporal cue [36].

**Figure 3:**
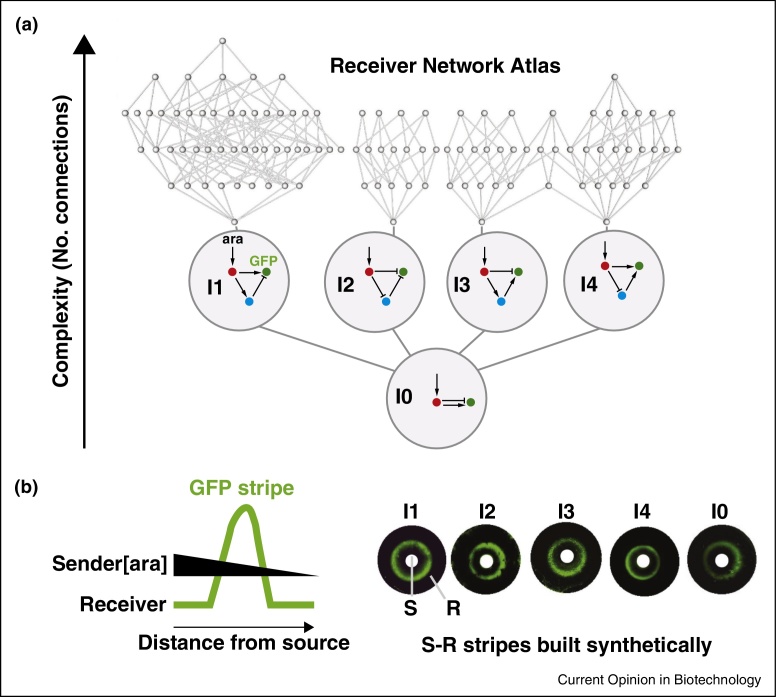
An systematic ‘network atlas’ approach finds all 3-node receiver networks that respond to a sender morphogen gradient by making a central stripe [37^••^]. **(a)** ∼3000 transcription activator-repressor networks were explored computationally, using 100,000 parameter sets, to see which would form stripes in response to a monotonic gradient of arabinose (ara). The green node (GFP) had to be OFF at *high* and *low* concentrations of arabinose (input to the red node) and ON at *middle* concentrations. The resulting 109 solutions (grey nodes) are organised by relative complexity, with four ‘stalactites’ indicating the minimal mechanisms for stripe formation (large circles). These mechanisms are all incoherent feedforward loops (I1,I2,I3,I4) and can be reduced even further to an archetypal 2-node network, *I*_zero_ (*I*_0_). **(b)** All minimal networks were constructed synthetically in *E. coli*. Lawns of bacteria on Petri dishes were tested against morphogen gradients from central paper disks (senders containing arabinose; white circles). All networks successfully exhibited stripe behaviour (green GFP rings). Importantly, the networks use distinct mechanisms and stripe-forming dynamics (i.e. they cannot be interconverted into each other merely by altering rate constants, etc.). The approach demonstrates the stripe forming capability of the entire incoherent network family.

Our own work took a systematic approach to explore band-patterning S–R networks [37^••^]. By exploring the 3-node network ‘design space’ exhaustively, we found that only a finite number of mechanisms can achieve stripe formation (Figure 3); we built all of these different mechanisms on a single flexible, synthetic biology scaffold, while developing an engineering method to ensure that networks function by a particular mechanism. Controlling mechanism precisely is essential to further progress in synthetic biology.

The examples above are based on one class of signalling agent: small diffusible chemical molecules. The information content of the molecules themselves is rather low, and the message conveyed is encoded in the amount of signal transferred. In an important conceptual leap, Ortiz and Endy are exploring methods of information transfer via DNA sequences encoded in the bacteriophage M13 [38]. Such methods have the potential for complex, high-content information transfer.

## Bidirectional communication: from artificial ecosystems to synchronised oscillators

Two-way communication, also employing diffusing signals between cells, has led to investigations of the computational potential of artificial ecosystems. For example, Brenner et al. achieved an AND-gate logic in *E. coli*, where signals from two complementary cell types had to accumulate to give an output, in the context of a cooperative microbial biofilm [39^•^]. A similar system, involving obligatory cooperation in yeast, explored the range of conditions that give rise to sustainable two-way codependence [40].

Predator-prey systems exhibit different two-way communication, involving negative feedback cycles, and have been built synthetically in *E. coli*, using microchemostats [41]. Synthetic ecosystems have even used bacterial and mammalian cell mixtures, leading to social behaviours like commensalism, ammensalism, mutualism, parasitism, and predator–prey oscillations [42].

Oscillatory systems, employing delayed negative feedback, are a favourite engineering target for synthetic biology, but a recent study elegantly employed an extra S–R layer to synchronise the oscillations in a population of bacterial cells [43]. An AHL system coupled cells to each other, ensuring that their oscillations occurred in phase. Coupling synthetic gene networks to intracellular S–R systems can lead to ‘sociability’ and reinforced population behaviours [44].

## Eukaryotic S–R systems: synthetic communication and patterning circuits

Synthetic biology in yeast, plants and mammals is sometimes seen as playing catch-up with its bacterial counterpart, but there is notable progress in engineering S–R systems. The first synthetic, eukaryotic cell-cell communication system was in yeast and employed a plant signalling hormone from *Arabidopsis* (cytokinin) to make positive feedback circuits [45^•^]. Two-way communication has also been engineered in mammalian cells, using L-tryptophan and acetaldehyde as signalling molecules [46^••^]. This system coupled the communication to a timed phenotype: the maturation of blood cells by growth factors.

Engineering networks inspired by embryonic developmental patterning is also a growing field within mammalian synthetic biology. Tetracycline gradient band-pass receiver systems [47] have been followed by fully genetically-encoded S–R systems [48]. In the latter study, diffusing activators and inhibitors, based on growth factors, were used to communicate and control gene expression over fields of cells, in 3D collagen cell culture. In principle, these components can be rewired to build many different pattern-forming network motifs [49,50].

## The next frontier: logic gates and distributed computing

Connecting sender–receiver systems in parallel yields combinatorial increases in complexity, and current efforts are exploring the possibility of building computational functions from communicating cells. An elegant trick to reduce the number of ‘wiring’ components for sending, receiving and processing signals, is to distribute tasks in consortia of different genetically-modified cells [51]. In this way, single cells perform simple robust functions, using a few well-characterised components, such as bacterial repressor proteins. The components can be reused in different logical gates or circuits — one per cell — so that the cell mixtures coordinate to process the information flow. Perhaps it is no accident that such work has come from researchers who were among the first to develop information theory in the context of genetic networks [52].

Cellular consortia have proved to be an efficient way of engineering complex tasks that are not easily solvable using single cells [42,53], including a *1-bit adder with carry function* [51]. There has also been significant progress in the amount of complexity that can be engineered within the single cells, with logic gates such as NOR being achieved in bacteria [53]. Importantly, NOR gates are ‘functionally complete’ and can be layered to achieve any computational operation; this opens up many engineering possibilities. For practical reasons, robustness in output can be increased at a population level by coupling the cell consortia using S–R systems with AHL signalling molecules.

The frontier of synthetic S–R systems is getting more and more diverse with the latest systems combining cell-cell communication and doped amyloid fibre formation [54]. Hence, communication systems are being coupled to self-assembling electrically conducting nanosystems, resulting in a convergence of biology, electronics and computation.

## Conclusion

Synthetic biology builds systems in order to understand them. Synthetic S–R systems are no exception, potentially giving insights into processes as diverse as spatiotemporal patterning, cellular computing through signalling, and neurological calculations. Moreover, the application of information theory puts biological communication on a quantitative footing, providing objective insights into how cell systems process signals. Such analyses could transform the way we think about the performance of real biological S–R systems, such as neurons in the brain.

Moving beyond the quantitation of information, key qualitative questions remain about how ‘meaning’ is transferred along with information. This is not merely an abstract question; synthetic biology can engineer reliable information transfer, but how would such systems encode or process higher order meaning, such as the difference between to ‘I must’ and ‘I want to’? Simple IF-THEN logic does not suffice. To harness essential features of biology, synthetic biologists somehow need to wire components to encode choice and reward, perhaps by including feedbacks in system resource allocation. We still do not know how to engineer higher order meaning, such as desire or fear. While information theory clearly has a part to play in increasing our engineering capability, we also need to develop a functional philosophy of meaning.

## References and recommended reading

Papers of particular interest, published within the period of review, have been highlighted as:

• of special interest
•• of outstanding interest
